# Dual Burden of Conflicts and Healthcare Collapse in the Democratic Republic of Congo: A Call for Action

**DOI:** 10.1002/puh2.70334

**Published:** 2026-08-13

**Authors:** Emile Cyubahiro, Darius Benimana

**Affiliations:** ^1^ Department of Medicine Gaziantep University Gaziantep Turkey; ^2^ Department of General Medicine and Surgery College of Medicine and Health Sciences University of Rwanda Kigali Rwanda

**Keywords:** armed conflicts, Democratic Republic of Congo, healthcare collapse, healthcare crisis, humanitarian crisis, insecurity

## Abstract

**Background:**

Healthcare is protected under international humanitarian law through principles such as civilian–combatant distinction and necessity. However, rising attacks on medical personnel and facilities in the Democratic Republic of Congo (DRC) represent serious violations of these laws, threatening uninterrupted care and undermining basic human rights.

**Objective:**

This narrative review synthesizes literature to highlight the compounded public health impact of ongoing conflict and health system collapse in the DRC to guide context‐sensitive interventions and policy actions for sustainable recovery.

**Methods:**

This narrative review synthesizes data up to 2025 from peer‐reviewed journals, accessed through PubMed, Google Scholar, World Health Organization (WHO), and World Bank databases, and other sources.

**Results:**

At the time of this study, ongoing conflict in the DRC, especially in the eastern provinces, has severely undermined healthcare access. Armed violence has led to the complete destruction of over 70 health facilities in North Kivu and partial destruction of 15 facilities in South Kivu, displacement of 7.3 million people, and disrupted vaccination campaigns, worsening cholera, Mpox, malaria, and measles outbreaks. Maternal and child mortality remain high due to insecurity, destroyed maternity centers, and limited antenatal care. Over 25% of the population faces severe food insecurity, with over 2 million children acutely malnourished. Insecurity, aid worker attacks, poor infrastructure, corruption, weak coordination, and donor fatigue hinder sustained recovery.

**Conclusion:**

The DRC faces a critical health crisis driven by conflict and healthcare system collapse. Urgent global action is required to protect health services, support frontline workers, and restore peace. Although some humanitarian responses have been implemented, sustainable, coordinated, context‐sensitive interventions and long‐term investments remain essential to uphold human rights and prevent further humanitarian catastrophe.

## Graphical abstract

The dual burden of conflict and healthcare attacks in the DRC has led to system collapse, resulting in the destruction of facilities, massive displacement, and the resurgence of deadly outbreaks. Urgent global action and coordinated, sustained investments are imperative to protect health services and prevent further catastrophe.

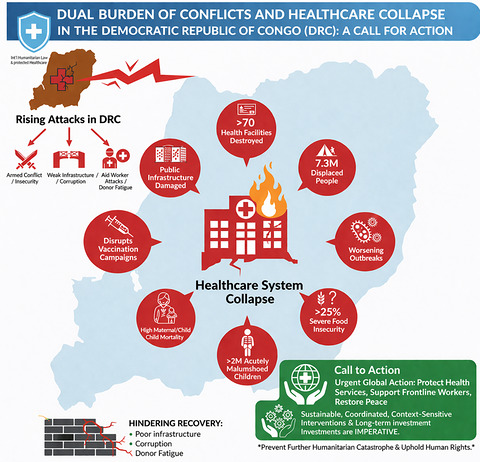

## 1 Introduction

The Democratic Republic of Congo (DRC), situated in Central Africa, is the second‐largest country in Africa and home to over 100 million people. The DRC has experienced a long history of conflict, political upheaval, and instability driven by colonial legacy, interethnic conflicts, resource competitions, and geopolitical interest [1, 2, 3]. Despite elections and peace agreements, instability persists, particularly in the eastern regions, which are home to over 140 armed groups [4]. Given its wealth in natural resources, including minerals and forests, the DRC is among the world's poorest nations [5]. Limited infrastructure makes it difficult to access healthcare services due to insufficient healthcare facilities [6]. Unfortunately, the political instability and insecurity are adding to the fragility of the country's health system.

The roots of this instability lie deep in history. Since 1996, the DRC has experienced multiple violent conflicts that have severely damaged its social and healthcare systems. Historical events, colonial legacies, interethnic disputes, resource competition, and geopolitical interests are the root causes of wars in the DRC [1, 2, 3]. The 1998–2003 conflict, known as Africa's World War, claimed 5.4 million deaths due to illness and a lack of access to healthcare brought on by ongoing wars from 1996 [7]. The eastern provinces continue to be the focal point of conflict despite the 2003 peace accord. The armed forces, like National Congress for the Defense of the People (CNDP) and later the March 23 Movement (M23), emerged from unsuccessful integration processes, with M23 briefly capturing the city of Goma in 2012 and re‐emerging with significant strength in 2022–2025 [8].

According to this analysis, the DRC is facing a dual burden resulting from ongoing armed conflict and a failing healthcare system. This further worsens the humanitarian situation, as escalating violence has displaced millions of people and caused thousands of deaths, further straining an already fragile healthcare system [9, 10]. The already fragile health infrastructure has been further weakened by the conflict. Armed violence has severely damaged healthcare infrastructure, leading to facility closures, displacement of health workers, and reduced access to essential services, whereas remaining hospitals are left overburdened and often inoperable [11, 12]. Civilians, particularly women and children, are disproportionately affected; widespread sexual violence, untreated psychological trauma, and ongoing obstacles to care, such as maternal services and sanitation services like water availability, further exacerbate the crisis [11, 13, 14]. Infectious disease outbreaks, including cholera, malaria, measles, and Mpox, have become increasingly difficult to control due to insecurity and disrupted health services [11, 15]. The situation is further worsened by patients fleeing isolation centers in search of safety, increasing the risk of disease transmission. Additionally, severe food insecurity affects more than 25% of the DRC's population, amid declining international humanitarian support [16].

Due to a significant reduction in donor financing, particularly from the United States (US), large humanitarian groups, such as the Danish Refugee Council, have been forced to reduce staff or leave the DRC entirely [3, 17, 18]. Meanwhile, $50 million has been requested by the World Health Organization (WHO) to continue emergency operations in the country [11, 19].

The convergence of violent conflict and a collapsing healthcare system has led to a surge in vaccine‐preventable diseases, maternal and child mortality, and mental health disorders [11]. These intertwined crises demand immediate global attention, collaborative efforts, and solidarity in finding coordinated long‐term solutions.

This narrative review synthesizes existing literature and recent developments to highlight the compounded public health impact of the DRC's dual burden of conflict and health system collapse. It aims to inform context‐sensitive interventions and policy actions for sustainable recovery.

## 2 Search Strategy

Articles and reports were selected on the basis of inclusion criteria focusing on the public health impacts of ongoing armed conflict and the collapse of the healthcare system in the DRC. These criteria included peer‐reviewed studies, humanitarian reports, and epidemiological data specific to the DRC, particularly in the heavily affected eastern provinces such as North and South Kivu. The review incorporated literature and data published between 2010 and 2025, with an emphasis on recent studies. Studies conducted outside the DRC, research not related to the intersection of conflict and health outcomes, and non‐English publications were excluded.

Database searches were conducted using PubMed, Google Scholar, WHO databases, and World Bank databases, supplemented by reports from other humanitarian organizations. The search strategy was structured around terms related to exposure (armed conflict and insecurity), outcomes (healthcare system collapse, healthcare crisis, and humanitarian crisis), and location (DRC). These terms were grouped into their respective categories, with the Boolean operator “AND” used to combine categories and “OR” used to link synonyms within each category. Additionally, manual searches of reference lists from relevant peer‐reviewed articles and institutional reports were conducted to identify further applicable literature.

## 3 Health System in Crisis

To maintain the DRC healthcare system and various humanitarian responses that were helping to combat HIV, malaria, and Mpox, as well as to ensure that food, water, and other necessities of life were available to marginalized families, the DRC, like other low‐ and middle‐income countries (LMICs), was receiving financial aid from the US [3, 11]. By delivering the biggest final share of the aid in 2024 through bilateral and humanitarian assistance, the US was the nation's largest contributor, accounting for over 70% of the country's humanitarian action with $1 billion [3]. Regretfully, 83% of the US Agency for International Development (USAID) contracts were formally terminated by the US in March 2025 [3]. Due to the cut of the funding and the incapacity to even support all health‐related projects in the conflict‐free environment, the country may experience more public health hardships.

Many people have fled the country, whereas others who could have contributed as trained personnel have also died [3, 11]. Healthcare facilities in conflict‐affected regions, particularly North Kivu, have been heavily damaged or rendered nonfunctional, resulting in overcrowded hospitals and reduced service capacity [3, 11, 20].

Through conflict and insecurity there is a cessation of healthcare services due to fear among healthcare workers [21]. It is also important to highlight the logistical problem of the destruction of the infrastructure, such as roads, electricity, and the cold chain system, which significantly impedes the delivery of essential medical supplies. This is likely to negatively affect several interventions that have already been implemented to guarantee health equity, such as the distribution of the vaccines against Mpox projects and other illnesses like malaria, measles, tuberculosis, and Ebola, which is likely to exacerbate the crisis [11].

## 4 Public Health Consequences of the Dual Crisis

The DRC is enduring an extended humanitarian catastrophe, with armed conflict and health system collapse reinforcing each other and producing devastating public health consequences. The resurgence of conflict, including the M23 rebellion and persistent violence from other armed groups, has compromised the already weak healthcare infrastructure, leading to widespread disease outbreaks and a dramatic increase in controllable and preventable deaths [3, 11].

Because of instability, displacement, and interrupted vaccination programs, disease outbreaks, including cholera, Ebola, measles, and Mpox, nearly all of which are human‐to‐human transmitted diseases, have become frequent and challenging to contain. For instance, the DRC saw the second‐largest Ebola outbreak in history between 2018 and 2020, which claimed over 2200 lives, whereas the Ministry of Health reported over 185,000 cases of cholera in 2023 [11, 22].

Water, sanitation, and hygiene conditions have deteriorated significantly, particularly in internally displaced people (IDP) camps. Overcrowding, unsafe water sources, and disrupted infrastructure have led to recurrent cholera outbreaks and other diarrheal diseases, increasing health risks among vulnerable populations [11].

Infectious disease surveillance and control remain weak, as reflected in the ongoing Mpox outbreak concentrated in eastern DRC [23]. In 2025, the outbreak prompted the WHO Director‐General to declare a public health emergency of international concern. Insecurity has disrupted response efforts, with patients fleeing isolation centers due to safety concerns, increasing the risk of further transmission [11].

Maternal and child mortality remains regrettably high, with 60.66 deaths per 1000 live births [24]. A decade‐long study found that hemorrhage, sepsis, and eclampsia are the leading causes of maternal deaths, most of which are preventable with timely intervention [25]. Insecurity contributes to delayed or absent attendance of pregnant women to the hospital as well as antenatal care. At the same time, infants face elevated risks of death from treatable illnesses like pneumonia and diarrhea that lead to increased infant mortality as well [26]. The lack of health professionals and the destruction of facilities, particularly maternity centers, mean women often give birth in unsafe conditions [25, 27].

Malnutrition, especially among children under five, is another major consequence of conflict and displacement. As of February 2025, during the ongoing conflict, one in four (or 25%) people in the region was already facing extreme hunger, with expectations of a worse situation if the insecurity and violence continue. Malnutrition and disease are closely linked. Malnourished individuals are less able to fight infections, whereas disease further worsens malnutrition [11]. Armed clashes disrupt agriculture and food distribution, exacerbating food insecurity in already fragile regions. Over 2 million children are estimated to be acutely malnourished in the DRC, with many in inaccessible areas where humanitarian services cannot reach due to security concerns [3, 11].

Repeated exposure to violence, displacement, and sexual abuse contributes significantly to the development of trauma, which is likely to create a large burden of untreated psychological disorders, hence seriously destructing mental health well‐being. Data collectors and frontline health workers in eastern DRC have reported post‐traumatic stress disorder (PTSD), anxiety, and burnout among both survivors and caregivers [27, 28]. Meanwhile, the country's mental health system remains severely underdeveloped, with only a few programs that can deliver psychosocial support, especially in rural areas.

WASH‐related illnesses, including diarrheal diseases and skin infections, are increasingly prevalent in IDP camps due to unsafe water sources and severe overcrowding [22, 29]. The United Nations International Children's Emergency Fund (UNICEF) and WHO have raised alarms about the deteriorating sanitation in IDP settlements, warning of a high risk of cholera resurgence [11, 22].

The ongoing violence has also made healthcare workers targets, further weakening the healthcare system. A recent study in eastern DRC revealed that over 80% of health workers had experienced direct violence, including assaults and threats, especially in Bukavu and Uvira [30]. This climate of fear has driven many to flee or cease practice, leading to a vacuum in care provision and a breakdown in continuity of essential health services [27, 30].

**FIGURE 1:**
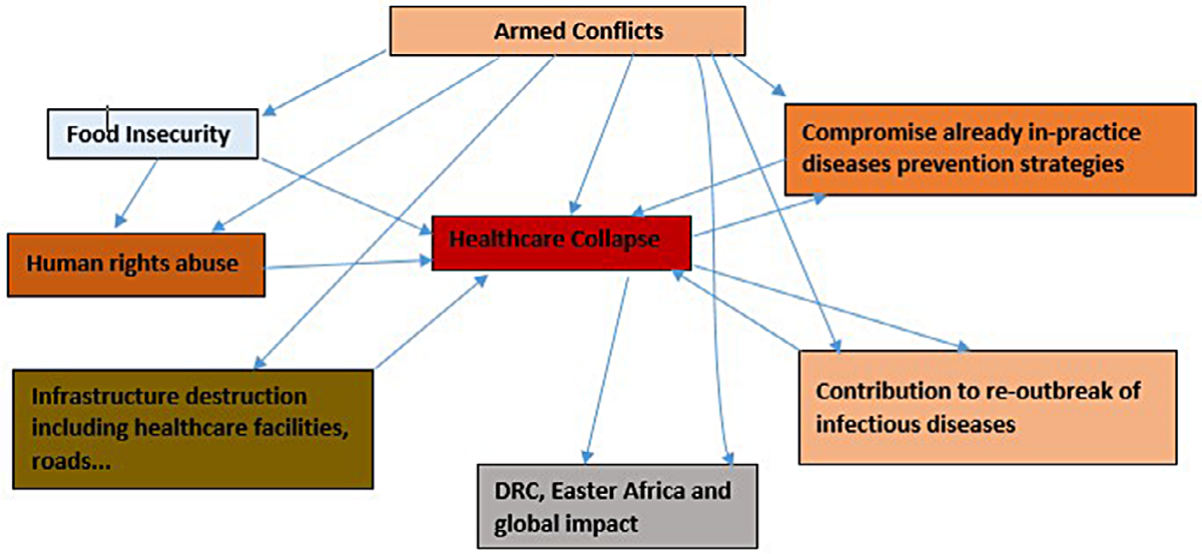
Dual burden of armed conflicts and healthcare collapse in the DRC. DRC, Democratic Republic of Congo.

The cumulative effect of this dual crisis is a population that is not only sick but also structurally unable to access the care they need. The eastern DRC tragedy is one of the most lethal and neglected humanitarian crises in the world, as WHO Director‐General has stated, necessitating both immediate action and long‐term system reconstruction [11]. Figure 1 displays cascading public health consequences of the armed conflicts in the DRC.

## 5 Barriers to Humanitarian Response

The humanitarian crisis in the DRC is complex and made worse by several obstacles that make it difficult to provide relief effectively. These difficulties include widespread insecurity, deliberate assaults on humanitarian workers, poor infrastructure, institutionalized corruption, and dwindling foreign assistance.

### 5.1 Insecurity Limiting Access to Rural Areas

Insecurity remains the primary barrier to healthcare delivery, especially in rural and conflict‐affected regions of eastern DRC. Ongoing violence has displaced more than 7.3 million people, many of whom have sought refuge in hard‐to‐reach areas with limited access to healthcare and humanitarian assistance [9, 11, 30]. The volatile security situation hinders the establishment of consistent humanitarian corridors to the problems, leaving vulnerable populations without essential services.

### 5.2 Attacks on Health and Aid Workers

Attacks on healthcare facilities and personnel are terribly common. In a mixed‐method study in eastern DRC in February 2021, 45.9% of healthcare workers reported direct exposure to violence, harassment, or threats while on duty [30]. These threats badly affect the morale and capacity of already overburdened health professionals [27]. For example, in 2023, two humanitarian workers were abducted, and their vehicles were destroyed in an attack in eastern DRC [31]. Such events not only put lives in danger but also hinder humanitarian organizations from maintaining their presence in critical areas, which, as a result, worsens the situation.

### 5.3 Poor Roads and Weak Infrastructure

Timely humanitarian assistance is further jeopardized by infrastructure constraints. It is logistically challenging to transport medical staff and supplies due to inadequate road networks, particularly in isolated areas. Recurrent flooding exacerbates the situation by cutting off important routes during emergencies [11, 32]. Due to weak health system infrastructure, many facilities lack water, electricity, or essential medical supplies [29].

### 5.4 Weak Coordination and Programs

Inadequate collaboration between government bodies, aid agencies, and community institutions often results in operational inefficiencies and overlapping interventions. Due to limited supervision, a substantial number of healthcare zones operate in isolation, which leads to inconsistent and fragmented healthcare provision [22]. This disorganization is further exacerbated by corruption and lack of transparency in resource allocation, which decrease interest for both local and international investment [28].

### 5.5 Donor Fatigue and Short‐Term Funding Cycles

Donor fatigue and unpredictable funding cycles create another major barrier. Despite the DRC being host of the world's most enduring humanitarian crises, it remains among the most underfunded emergencies globally. Despite an alarming escalation in needs due to the ongoing humanitarian crisis, the US has recently diminished humanitarian aid through the region and has been getting large amounts of its financial support [3]. Short‐term grants also do not ensure sustainable interventions, as they are often abruptly terminated without building long‐term resilience or health systems strengthening [11, 32].

Taken together, these obstacles not only delay the provision of lifesaving support but also increase vulnerability, exacerbating public health emergencies such as measles, cholera, malnutrition maternal health, and Mpox, among others.

## 6 Current Interventions and Programs

Despite the pervasive humanitarian crisis, various humanitarian programs have been operating in the DRC toward provision of humanitarian response. Table 1 summarizes major health‐related programs in the DRC, their operational focus, and key challenges or limitations they face in their operations.

**TABLE 1:** Major health‐related programs in the Democratic Republic of Congo (DRC), their operational focus, and key challenges or limitations.

Organization/Program	Focus area	What they do	Challenges/Limitation
United Nations Organization Stabilization Mission for the DRC (MONUSCO)	Peacekeeping and protection	Protects civilians, hospitals, and aid workers amid ongoing armed conflict [32]	Limited geographic reach; loss of trust from the community; and inability to prevent all attacks [32]
WHO Health Cluster	Emergency health conditions	Leads outbreak coordination and emergency medical response [33]	Breakdown of coordination; restricted in conflicted‐heavy zones [33]
Global Alliance for Vaccines and Immunization (GAVI) and Global Fund	Vaccination and infectious disease control	Provides vaccines for measles, malaria, HIV, and COVID‐19 [34]	Supply chains disruption by insecurity; reliance on external funds [34]
United Nations Population Fund (UNFPA)	Maternal and reproductive health	Delivers birth kits, trains midwives, offers gender‐based violence services in crisis zones [35]	Cultural beliefs, stigma around gender‐based violence (GBV), and infrequent access in rural areas [35]
USAID Integrated Health Program (IHP)	Strengthening health system	Constructs facilities, trains providers, improves service delivery [36]	Limited reach, lack of decentralized activities delays in provincial rollout [36]
Community Health Worker (CHW) initiatives	Community‐based healthcare	Local healthcare workers provide basic care and health education [37]	Low or no payment; violence; limited tools and medicine [37]

*Note:* As the table above displays, regardless of significant contributions, many programs face challenges in reaching areas most in need of interventions, funding, and coordination constraints. However, by improving local partnerships, decentralized services, and focusing on community‐based solutions, these actors can better adapt to the fragile situation of the DRC and expand their reach.

## 7 Recommendations

Healthcare personnel in eastern DRC experience daily threats, including physical violence, abductions, and the destruction of medical infrastructure [28, 30]. As was done in some areas of the world like Syria and Sudan, establishing protected health zones under international humanitarian law could safeguard both workers and facilities [11]. Community‐based protection strategies that engage local leaders in co‐designing safety protocols for health workers have also shown success in rural South Kivu [27].

Training and providing stipends to the CHWs and the use of mobile digital health tools can substantially increase their capacity and safety [38]. CHWs remain the backbone of health service delivery in insecure, rural areas of the DRC. In spite of playing a central role, they often work undertrained and unsafe [39, 40, 41]. The USAID IHP has witnessed success in this area, showing improved maternal and child health outcomes in areas where CHWs were adequately trained and kept with resources [25].

Integrating mental and psychological support (MHPSS) services into primary healthcare systems is crucial, particularly for women, children, and survivors of sexual violence [23]. Years of chronic violence, displacement, and trauma have led to widespread mental health disorders in conflict‐affected populations [22, 42].

In conflict zones, the breakdown of transport and cold chain systems severely limits vaccine delivery and medicine distribution [29]. Solar‐powered cold chain systems and drone‐based deliveries have demonstrated success in some countries like Rwanda and Uganda. For instance, the blood product drone‐delivery implementation in Rwanda has significantly saved time and wastage and could be adapted to the DRC's topography [43, 44, 45]. However, creating a contingency logistics plan that would be adapted once the supply chain is disrupted to ensure timely delivery of services is important. The partnership with local organizations is also vital to ensure better distribution networks and storage facilities.

Former health interventions mostly fail when they do not take into consideration local conflict dynamics. Conflict‐sensitive strategies involve relating health priorities with local governance structures, ethnic dynamics, and cross‐border conflict risks [26, 27]. Interventions must be tailored to local contexts and community needs.

Short‐term aid cycles and donor fatigue undermine sustainable health outcomes [46]. Locally led health development initiatives, such as the Panzi Foundation's model combining hospital care, community outreach, and legal support for GBV survivors, illustrate the power of long‐term, integrated approaches [47]. International donors and agencies should prioritize multiyear flexible funding, local leadership, and capacity‐building to ensure the long‐lasting interventions.

## 8 Limitations and Future Directions

This review primarily relied on secondary data, including previously published reports and reviews, which may limit the availability of real‐time data and an accurate assessment of health threats arising from the conflicts. Furthermore, potential biases in humanitarian reports could affect the generalizability of findings and the applicability of recommendations for public health systems. The lack of primary studies necessitated a narrative synthesis rather than a systematic meta‐analysis, limiting replicability and the ability to generate robust evidence to guide health policy. Future research should prioritize the collection of primary data from affected sites, including retrospective analyses of health facility records, to identify specific healthcare complications resulting from conflicts. Such studies would provide evidence‐based guidance for targeted interventions and effective humanitarian responses in the region.

## 9 Conclusion

The DRC is experiencing a severe humanitarian crisis driven by the intersection of prolonged armed conflict and systemic healthcare collapse. This dual burden has created a reinforcing cycle in which insecurity damages health infrastructure, disrupts essential services, and contributes to the resurgence of preventable diseases, whereas a weakened health system increases population vulnerability and mortality.

The consequences are extensive, including widespread displacement, rising infectious disease outbreaks, persistent food insecurity, and high maternal and child mortality. These challenges are further intensified by attacks on healthcare workers, fragile infrastructure, weak governance, and declining international support, all of which hinder both immediate response and long‐term recovery.

Addressing this crisis requires more than short‐term humanitarian assistance. It calls for sustained, coordinated strategies that protect healthcare systems, strengthen local capacity, and ensure equitable access to essential services. Investment in resilient infrastructure, community‐based care, and long‐term funding mechanisms is essential.

Ultimately, improving health outcomes in the DRC is closely linked to achieving peace and stability. Without sustained international commitment to address both conflict and health system fragility, the crisis will persist. The situation represents not only a public health emergency but also a critical issue of human rights and global responsibility.

## Author Contributions

**Emile Cyubahiro**: conceptualization, writing – original draft, writing – review and editing, data curation. **Darius Benimana**: writing – review and editing, data curation, supervision.

## Ethics Statement

The authors have nothing to report.

## Consent

The authors have nothing to report.

## Conflicts of Interest

The authors declare no conflicts of interest.

## Data Availability

Data sharing is not applicable to this article as no datasets were generated or analyzed during the current study.
